# Repeatability quantification of brain diffusion-weighted imaging for future clinical implementation at a low-field MR-linac

**DOI:** 10.1186/s13014-024-02424-7

**Published:** 2024-03-06

**Authors:** Moritz Rabe, Olaf Dietrich, Robert Forbrig, Maximilian Niyazi, Claus Belka, Stefanie Corradini, Guillaume Landry, Christopher Kurz

**Affiliations:** 1 Department of Radiation Oncology, LMU University Hospital, LMU Munich, Munich, Germany; 2 Department of Radiology, LMU University Hospital, LMU Munich, Munich, Germany; 3 Institute of Neuroradiology, LMU University Hospital, LMU Munich, Munich, Germany; 4 German Cancer Consortium (DKTK), Partner Site Munich, a Partnership Between DKFZ and LMU University Hospital Munich, Munich, Germany; 5 Bavarian Cancer Research Center (BZKF), Munich, Germany; 6 Department of Radiation Oncology, University of Tübingen, Tübingen, Germany

**Keywords:** Diffusion-weighted imaging, Apparent diffusion coefficient, MR-linac, Low-field MRI, MR-guided radiotherapy, Functional imaging, Repeatability, Intracranial radiotherapy

## Abstract

**Background:**

Longitudinal assessments of apparent diffusion coefficients (ADCs) derived from diffusion-weighted imaging (DWI) during intracranial radiotherapy at magnetic resonance imaging-guided linear accelerators (MR-linacs) could enable early response assessment by tracking tumor diffusivity changes. However, DWI pulse sequences are currently unavailable in clinical practice at low-field MR-linacs. Quantifying the in vivo repeatability of ADC measurements is a crucial step towards clinical implementation of DWI sequences but has not yet been reported on for low-field MR-linacs. This study assessed ADC measurement repeatability in a phantom and in vivo at a 0.35 T MR-linac.

**Methods:**

Eleven volunteers and a diffusion phantom were imaged on a 0.35 T MR-linac. Two echo-planar imaging DWI sequence variants, emphasizing high spatial resolution (“highRes”) and signal-to-noise ratio (“highSNR”), were investigated. A test–retest study with an intermediate outside-scanner-break was performed to assess repeatability in the phantom and volunteers’ brains. Mean ADCs within phantom vials, cerebrospinal fluid (CSF), and four brain tissue regions were compared to literature values. Absolute relative differences of mean ADCs in pre- and post-break scans were calculated for the diffusion phantom, and repeatability coefficients (RC) and relative RC (relRC) with 95% confidence intervals were determined for each region-of-interest (ROI) in volunteers.

**Results:**

Both DWI sequence variants demonstrated high repeatability, with absolute relative deviations below 1% for water, dimethyl sulfoxide, and polyethylene glycol in the diffusion phantom. RelRCs were 7% [5%, 12%] (CSF; highRes), 12% [9%, 22%] (CSF; highSNR), 9% [8%, 12%] (brain tissue ROIs; highRes), and 6% [5%, 7%] (brain tissue ROIs; highSNR), respectively. ADCs measured with the highSNR variant were consistent with literature values for volunteers, while smaller mean values were measured for the diffusion phantom. Conversely, the highRes variant underestimated ADCs compared to literature values, indicating systematic deviations.

**Conclusions:**

High repeatability of ADC measurements in a diffusion phantom and volunteers’ brains were measured at a low-field MR-linac. The highSNR variant outperformed the highRes variant in accuracy and repeatability, at the expense of an approximately doubled voxel volume. The observed high in vivo repeatability confirms the potential utility of DWI at low-field MR-linacs for early treatment response assessment.

**Supplementary Information:**

The online version contains supplementary material available at 10.1186/s13014-024-02424-7.

## Background

Magnetic resonance imaging (MRI)-guided radiotherapy with MR-guided linear accelerators (MR-linacs) enables online adaptive treatments based on daily acquired MRI datasets and gated beam delivery with the aid of real-time cine MRI for enhanced accuracy compared to conventional image-guided radiotherapy [1, 2]. While MR-linacs are predominantly employed for irradiating mobile tumors in the abdominal, thoraic, or pelvic region [3, 4], the high soft tissue contrast of MRI and the potential for online treatment plan adaptation also allows intracranial treatments with increased precision. Intracranial treatments at MR-linacs can be performed with smaller target volumes and reduced neurotoxicity compared to treatments at conventional linacs [2, 5–7]. Another key feature of MR-linacs, not yet fully exploited in clinical practice, is the technical feasibility of functional MRI. Functional images acquired throughout treatment could enable early treatment response assessment and biologically-guided radiotherapy [1, 2, 8, 9].

Diffusion-weighted imaging (DWI) is being considered as one of the most promising functional imaging techniques applicable to MR-linac systems [9, 10]. DWI provides quantitative information about the tumor cellularity and cell membrane integrity. When integrated into radiotherapy workflows, DWI can enhance tumor characterization and delineation, treatment outcome prediction, and early response assessment across diverse tumor sites, such as the prostate, cervix, rectum, head and neck, and brain [11–18]. At MR-linacs, regular DWI throughout the treatment course enables early response assessment by monitoring changes in tumor diffusivity, quantified in apparent diffusion coefficient (ADC) maps [9, 10, 12, 15, 19]. Within the domain of intracranial radiotherapy, multiple studies have validated the utility of ADC maps derived from DWI as early prognostic imaging biomarker [15, 20, 21]. This has sparked high interest within the MR-guided radiotherapy research community to integrate DWI into the clinical routine at high- and low-field MR-linac systems [9, 22].

Today, no DWI pulse sequence is clinically available at the 0.35 T ViewRay MRIdian MR-linac. DWI at the MRIdian was initially described for the tri-Cobalt-60 radiotherapy system [23], utilizing echo-planar imaging (EPI) and turbo spin echo (TSE)-based diffusion-weighted sequences with high ADC accuracy and reproducibility in phantoms [24, 25]. In vivo DWI at the ViewRay MRIdian tri-Cobalt-60 system was explored for head and neck cancer, sarcoma, glioblastoma, and rectal cancer [24–27], with the primary goal of monitoring ADCs within the tumor and normal tissue throughout treatment. Following the upgrade of the MRIdian to an MR-linac [28], both EPI [29] and TSE [30] based DWI pulse sequences were investigated with diffusion phantoms at these systems. However, ADC inaccuracies were reported for the EPI sequence [29], and severe image artifacts were observed for the TSE sequence [30]. More recently, Weygand et al. demonstrated the use of DWI with an EPI sequence at the MRIdian MR-linac system with excellent ADC accuracy and repeatability for a NIST traceable diffusion phantom and first reported on the in vivo application of DWI at an MRIdian MR-linac for five sarcoma patients [31].

The findings presented by Weygand et al. hold promise for the integration of DWI into the clinical workflow of the MRIdian MR-linac. However, the optimal parameters for DWI pulse sequences differ across various anatomical sites, necessitating adjustments before applying them to brain cancer patients [12, 13]. Moreover, high in vivo repeatability of ADCs is crucial for conducting longitudinal evaluations of ADC changes for early response assessments, and quantification of the repeatability of a DWI pulse sequence on a specific system is essential for establishing appropriate action levels in a biologically-guided radiotherapy approach, ensuring the distinction between measurement uncertainties and true biomarker changes [32–35]. Despite this importance, the in vivo repeatability of ADC maps acquired with DWI pulse sequences on low-field MR-linac systems has not yet been investigated.

To address these research gaps, our study aimed to quantify the repeatability of ADC measurements derived from an EPI DWI pulse sequence adapted for brain imaging with a diffusion phantom and a volunteer cohort on a 0.35 T MR-linac system. Additionally, we compared ADCs in different liquids within the diffusion phantom and various regions-of-interest (ROIs) within brain tissue to literature values.

## Methods

The present study aimed to assess the repeatability of ADC measurements derived in a diffusion phantom and in brains of healthy volunteers. The diffusion phantom, previously described by Dietrich et al. comprises four glass vials with a 68 mm diameter, containing liquids of varying diffusivities, namely water, acetone, polyethylene glycol (PEG), and dimethyl sulfoxide (DMSO) [36]. The volunteer cohort consisted of eleven individuals (six male and five female), with a median age of 29 years (range 23–38 years). The study was conducted according to the guidelines of the Declaration of Helsinki and was approved by the Institutional Review Board of the Medical Faculty of the LMU University Hospital, LMU Munich (reference number: 22-0954). Informed consent was obtained from all volunteers participating in the study.

Imaging was conducted at a 0.35 T MRIdian MR-linac system (ViewRay Inc., Oakwood Village, OH, USA) [28] at the Department of Radiation Oncology at the LMU University Hospital (LMU Munich). Prior to image acquisition, the treatment delivery system and MRI scanner were decoupled to operate the MR-linac in quality assurance mode to allow for modification of the sequence parameters, and the gantry angle was set to 0°. The diffusion phantom was stored in the treatment room ahead of time to ensure thermal equilibrium. For both diffusion phantom and volunteer scans, the head and neck receiver coils of the system were used, following the setup procedure described by Konnerth et al. but without using a thermoplastic mask [37].

### Diffusion-weighted imaging sequence optimization

Before systematically imaging volunteers following the scanning protocol described below, the parameters of the DWI pulse sequence were optimized for brain imaging for one volunteer. For this purpose, a prototype single-shot EPI DWI pulse sequence provided by the vendor was adapted in terms of b-values, number of averages, spatial resolution, field-of-view, repetition time, and bandwidth, all while simultaneously considering image quality, spatial resolution, and acquisition time. Two sequence variants were chosen for further investigation: one with a focus on a high spatial resolution (“highRes”), and the other with a focus on a high signal-to-noise ratio (“highSNR”). The respective sequence parameters are summarized in Table 1. The acquisition time for both sequence variants was approximately 6.5 min. The rationale behind this was to ensure that the DWI scan could be obtained within the timeframe allocated for reviewing and adapting the treatment plan between the acquisition of the daily setup MRI scan and the initiation of treatment delivery, thus avoiding any extension of the overall treatment fraction time in clinical practice.

**Table 1:** Parameters of the two investigated EPI DWI sequence variants

Sequence variant	High resolution (“highRes”)	High SNR (“highSNR”)
b-values (s/mm^2^)	0, 100, 250, 500, 800	0, 100, 250, 500, 800
Acquisition time (min:s)	6:36	6:20
Slice thickness (mm)	5	7
Number of slices	20	14
Acquisition matrix	90 × 90	80 × 80
In-plane acquisition matrix resolution (mm^2^)	3.0 × 3.0	3.5 × 3.5
In-plane image resolution after zero-filling interpolation (mm^2^)	1.5 × 1.5	1.75 × 1.75
Field-of-view (mm^3^)	270 × 270 × 100	280 × 280 × 98
Number of averages	9	11
TR/TE (ms)	3300/110	2600/110
Bandwidth (Hz/pixel)	1355	1359
Flip angle	90°	90°

For both sequence variants, axial diffusion-weighted images at five different diffusion weightings (b-values) were acquired (0, 100, 250, 500, 800 s/mm^2^), where the diffusion gradient was subsequently applied in the three cardinal directions (phase, read, slice). While the acquisition times and the field-of-view were similar for both variants, the main differences were in the number of averages (9 for highRes vs. 11 for highSNR), slice thickness and number (20 slices of 5 mm versus 14 slices of 7 mm), and in-plane voxel size (acquisition matrix voxel size of 3.0 × 3.0 mm^2^ versus 3.5 × 3.5 mm^2^). For both variants, zero-filling interpolation was applied before image reconstruction to obtain an image in-plane resolution of 1.5 × 1.5 mm^2^ and 1.75 × 1.75 mm^2^ for highRes and highSNR, respectively.

The remaining ten volunteers were scanned with these two sequence variants in a test–retest study [32, 34, 35], following the scanning protocol described below.

### Data acquisition and imaging workflow

A test–retest study with an intermediate out-of-scanner break and repositioning was conducted to assess the repeatability of ADC measurements within the diffusion phantom and ten volunteers. During initial positioning at the MR-linac, the position of the projected virtual isocenter indicated by lasers outside of the scanner bore [28] was marked on adhesive tape attached to the phantom or volunteers’ foreheads, and the respective treatment couch positions were recorded.

After setup, the same scanning protocol was followed for both the phantom and volunteers. First, a 3D-MRI dataset was acquired with a clinical balanced steady-state free precession (bSSFP) sequence (TrueFISP; sagittal slices; slice thickness: 1.5 mm; in-plane resolution: 1.49 × 1.49 mm^2^; TR/TE: 3.84/1.92 ms; bandwidth: 532 Hz/pixel; flip angle: 60°; field-of-view (LR × AP × SI): 216 × 268 × 280 mm^3^). This was followed by acquisition of the two DWI sequence variants detailed above (Scan 1; test). Subsequently, the phantom or volunteer were moved out of the scanner bore for a break between scans of at least 5 min. For this, the phantom was removed from, and volunteers were instructed to step off the treatment couch. After the break, the phantom or volunteers were repositioned by moving the treatment couch to the same position as during initial scanning and with the aid of the laser positioning system and marked positions. Subsequently, another 3D-MRI dataset and the two DWI sequence variants were acquired (Scan 2; retest). All acquired data were exported in DICOM format for offline analysis.

### Apparent diffusion coefficient map reconstruction

All ADC maps were reconstructed offline with an in-house Python script (Python 3.8.10). The geometric mean values of the direction-specific diffusion-weighted images were calculated and fitted pixel-wise with the Python package scipy.optimize.minimize (scipy version 1.3.3; optimizer L-BFGS-B) using the monoexponential function (with two fit parameters):

1 $$S\left( b \right) = S_{0} \exp \left( { - b \cdot {\text{ADC}}} \right),$$

with the signal *S*_0_ at *b* = 0 and *S*(*b*) at b-value *b*, and the ADC. This resulted in four ADC maps for the diffusion phantom and for each volunteer (two sequence variants for both Scan 1 and Scan 2).

### ADC accuracy and repeatability analysis

For the diffusion phantom and each volunteer, the duration of the outside-scanner break, defined as the time span between the end of sequence variant highSNR in Scan 1 and start of imaging in Scan 2, was calculated.

For analysis of the ADCs, all 3D-MRI datasets and ADC maps were imported into a research version of the treatment planning system RayStation 10B (version 10.1.100.0; RaySearch Laboratories, Stockholm, Sweden). The pre- and post-break 3D-MRI datasets were rigidly registered using the automatic intensity-based rigid registration with correlation coefficient as image similarity measure implemented in the treatment planning system. The results of the registration were visually inspected in overlay plots. The resulting translation vectors and rotations were applied to the ADC maps acquired after the break to map all ADC maps to the same frame-of-reference.

Contouring was performed on the 3D-MRI dataset of Scan 1. For the diffusion phantom, the four vials were contoured, and the contours were contracted by 7 mm for sampling the ADCs in the center of the four liquids contained in the vials (water, DMSO, acetone, and PEG). For the volunteers, the cerebral ventricles were segmented, and the contours were contracted by 2 mm to sample the ADCs within the cerebrospinal fluid (CSF). Additionally, four cylindrical regions-of-interest (ROIs) with a 1 cm radius, a 2.5 cm height, and a volume of 7.9 cm^3^ located to the left (ROI_left_) and right (ROI_right_) of the ventricles and in the posterior right brain hemisphere (ROI_post_) and anterior left hemisphere (ROI_ant_) were defined in regions of relatively homogenous image contrast as observed on the 3D-MRI dataset. All structures were propagated to the four registered ADC maps for the diffusion phantom and each volunteer, respectively.

The average ADCs (mean ± 1σ) within each of the ROIs on each dataset were extracted and compared to literature values. Concerning the diffusion phantom, literature values were retrieved from a study in which the identical diffusion phantom was scanned at a diagnostic 1.5 T MRI scanner at a room temperature of 24 °C with different sequences [36]. The range of ADCs measured with a single-shot EPI DWI pulse sequence in three diffusion directions (read, phase, slice) was considered for comparison. For evaluation of the ADCs in the CSF and the cylindrical ROIs within the volunteers’ brains, reference values were obtained from a publication quantifying the ADCs in various regions of the brains of healthy volunteers [38]. As the cylindrical ROIs contained mixtures of white and gray matter tissue, the overall range of ADCs reported for these two tissue types was considered.

To assess the repeatability of the measurements of the mean ADC in the ROIs in the diffusion phantom, the absolute relative deviation *Δ* (in percent) was calculated as the absolute difference of the mean ADCs measured in Scan 1 (ADC_1_) and Scan 2 (ADC_2_) relative to their mean value [39]:

2 $$\Delta = \frac{{\left| {{\text{ADC}}_{1} - {\text{ADC}}_{2} } \right|}}{{{\text{mean}}\left( {{\text{ADC}}_{1} , {\text{ADC}}_{2} } \right)}} \cdot 100\% .$$

The deviation *Δ* was calculated for both sequence variants for each ROI of the diffusion phantom (water, DMSO, acetone, and PEG).

Following the Quantitative Imaging Biomarkers Alliance (QIBA) recommendations and definitions [32, 34, 35], the repeatability coefficient (RC) of the mean ADCs, measured in a test–retest scheme, was calculated for each ROI for the volunteers (CSF, ROI_left_, ROI_right_, ROI_post_, ROI_ant_). The RC is a metric for the precision and quantifies the range within which 95% of differences between measurements of a biomarker under repeatability conditions within the same subject are expected to fall due to inherent measurement uncertainties [32]. For large sample sizes, the RC for repeated measurements of *N* subjects is defined as [33, 35, 40–42]:

3 $${\text{RC}} = 1.96 \cdot \sqrt 2 \cdot {\text{wSD}} = 1.96 \cdot \sqrt 2 \cdot \sqrt {\frac{1}{N}\mathop \sum \limits_{i = 1}^{N} \sigma_{i}^{2} } ,$$

with the within-subject standard deviation wSD of the mean ADC within the ROI, the number of volunteers *N*, and the within-subject variances $\sigma_{i}^{2}$. With two measurements (test and retest) for a given ROI and volunteer *i*, with mean values of ADC_*i,*1_ (Scan 1) and ADC_*i,*2_ (Scan 2), the within-subject variance is (ADC_*i,*1_-ADC_*i,*2_)^2^/2, and the RC can be written as:

4 $${\text{RC}} = 1.96 \cdot \sqrt {\frac{{\mathop \sum \nolimits_{i = 1}^{N} \left( {{\text{ADC}}_{i,1} - {\text{ADC}}_{i,2} } \right)^{2} }}{N}} .$$

For small sample sizes (*N* < 30), the factor of 1.96 needs to be adjusted, by using the critical value *t*_df_ of the Student’s *t*-distribution with *N* − 1 degrees of freedom (df) at a 95% confidence level, instead. Consequently, for this study, the RC calculation was adjusted accordingly:

5 $${\text{RC}} = t_{{{\text{df}}}} \cdot \sqrt {\frac{{\mathop \sum \nolimits_{i = 1}^{N} \left( {{\text{ADC}}_{i,1} - {\text{ADC}}_{i,2} } \right)^{2} }}{N}} .$$

The 95% confidence intervals [RC_L_, RC_U_] (CIs) for the RC were calculated using the 97.5th and 2.5th percentile values of a $\chi^{2}$ distribution. The lower and upper limits of the CIs, RC_L_ and RC_U_, are given by [33]:

6 $${\text{RC}}_{{\text{L}}} = {\text{RC}} \cdot \sqrt {\frac{{{\text{df}}}}{{\chi_{{{\text{df}}}}^{2} \left( {0.975} \right)}}} .$$

and

7 $${\text{RC}}_{{\text{U}}} = {\text{RC}} \cdot \sqrt {\frac{{{\text{df}}}}{{\chi_{{{\text{df}}}}^{2} \left( {0.025} \right)}}} .$$

Furthermore, the relative repeatability coefficient (relRC; in %) was calculated [35, 42]:

8 $$\begin{aligned} {\text{relRC}} & = t_{{{\text{df}}}} \cdot \sqrt 2 \cdot {\text{wCV}} \cdot 100\% = t_{{{\text{df}}}} \cdot \sqrt 2 \cdot \sqrt {\frac{1}{N}\mathop \sum \limits_{i = 1}^{N} \frac{{\sigma_{i}^{2} }}{{\mu_{i}^{2} }}} \cdot 100\% \\ & = t_{{{\text{df}}}} \cdot \sqrt {\frac{1}{N}\mathop \sum \limits_{i = 1}^{N} \frac{{\left( {{\text{ADC}}_{i,1} - {\text{ADC}}_{i,2} } \right)^{2} }}{{{\text{mean}}\left( {{\text{ADC}}_{1} , {\text{ADC}}_{2} } \right)^{2} }}} \cdot 100\% , \\ \end{aligned}$$

with the within-subject coefficient of variation wCV, and the mean value $\mu_{i}$ of ADC_*i,*1_ (Scan 1) and ADC_*i,*2_ (Scan 2). The 95% CIs of the relRC were calculated analogously to the CIs of the RC.

The RC and relRC values with the respective CIs were calculated for the CSF and each cylindrical ROI separately, using a critical value of *t*_9_ = 2.262 (*N* = 10 volunteers; df = *N* − 1 = 9). For better comparability with RCs reported in the literature, these metrics were additionally calculated for all four cylindrical ROIs within the brain tissue combined, using a critical value of *t*_39_ = 2.023 (4 ROIs for each volunteer; df = 40 − 1 = 39).

Additionally, Bland–Altman plots for the mean ADCs measured within the ROIs in the volunteers were generated for the CSF, and for the four cylindrical ROIs combined, and respective biases and limits of agreement (LoAs) at 95% confidence were determined [41].

## Results

### Diffusion phantom

The ambient temperature in the MR-linac room during the diffusion phantom scans was recorded at 22.4 °C. The break duration was 7.5 min. The ROIs used for evaluating the ADCs had volumes of 115 cm^3^, 118 cm^3^, 103 cm^3^, and 113 cm^3^ for water, DMSO, acetone, and PEG, respectively.

The measured ADCs (mean ± 1σ) for the four ROIs, across the two scans and sequence variants are reported in Table 2, alongside absolute relative deviations, and literature value ranges. Except for PEG, the ADCs obtained with the highSNR variant were larger and closer to the literature values compared to the highRes variant. Considering the mean ADC, averaged over Scans 1 and 2, the relative deviations from the lower end of the reported literature value ranges were − 18%, − 15%, − 86%, and − 2% for highRes, and − 10%, − 11%, − 76%, and − 5% for highSNR, for water, DMSO, acetone, and PEG, respectively. Thus, the mean ADCs for acetone differed the most from the literature values (which is discussed below). The standard deviation of ADCs within the ROIs ranged between 2 and 8% of their mean values for water, DMSO, and PEG, and up to 30% for acetone.

**Table 2:** Summary of diffusion phantom results

ROI	highRes			highSNR			Literature (24 °C)
	Scan 1	Scan 2	*Δ*	Scan 1	Scan 2	*Δ*	
Unit	10^–6^ mm^2^/s	10^–6^ mm^2^/s	%	10^–6^ mm^2^/s	10^–6^ mm^2^/s	%	10^–6^ mm^2^/s
Water	1812 ± 89	1812 ± 89	0.0	1999 ± 71	2003 ± 72	0.2	2216–2285
DMSO	612 ± 46	617 ± 51	0.8	641 ± 39	646 ± 39	0.8	719–798
Acetone	481 ± 144	484 ± 146	0.6	870 ± 196	843 ± 195	3.2	3514–3617
PEG	328 ± 13	330 ± 13	0.6	321 ± 7	322 ± 7	0.3	337–378

The ADCs (mean ± 1σ), averaged over all voxels within each ROI are reported along with the absolute relative deviation *Δ* of the mean values between the two repeated scans for both DWI sequence variants and literature value ranges [36]

When assessing the repeatability of the mean ADCs between Scan 1 and 2, deviations smaller than 1% were attained for both sequence variants, except for acetone with highSNR, for which a value of 3.2% was measured (Table 2). No clear difference in repeatability was observed between the two sequence variants.

### Volunteers

**Fig. 1:**
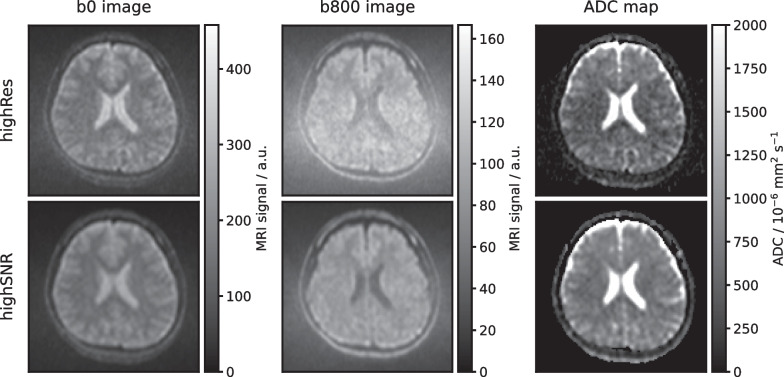
Images at different b-values and ADC maps for one volunteer. An axial slice of the b0 image (left column), b800 image (center), and respective ADC maps (right) are shown for the highRes (top row) and highSNR (bottom) sequence variants. The b0 and b800 image views have different window and level settings to maximize image contrast. Due to different slice thicknesses and positions of the two sequence variants, the depicted anatomy differs slightly

In Fig. 1, axial slices of representative b0 and b800 images (representing the lowest and highest acquired b-values) along with the corresponding reconstructed ADC maps are displayed for a volunteer for both sequence variants. The depicted anatomy slightly differs due to the different slice thicknesses and positions between the two sequence variants. The voxel size in the acquisition matrix (prior to image reconstruction) was 45 mm^3^ for the highRes and 86 mm^3^ for the highSNR sequence variant, thus 1.9 times larger for the latter. The difference in spatial resolution is most evident in the b0 images in Fig. 1, where the highSNR variant appears blurrier compared to the highRes variant. The higher spatial resolution for the highRes variant led to a notably more pronounced noise level in the b800 images compared to the highSNR variant. Additionally, the ADCs depicted in Fig. 1 appear lower for the highRes variant compared to the highSNR variant, particularly noticeable in the brain tissue on either side of the ventricles.

**Fig. 2:**
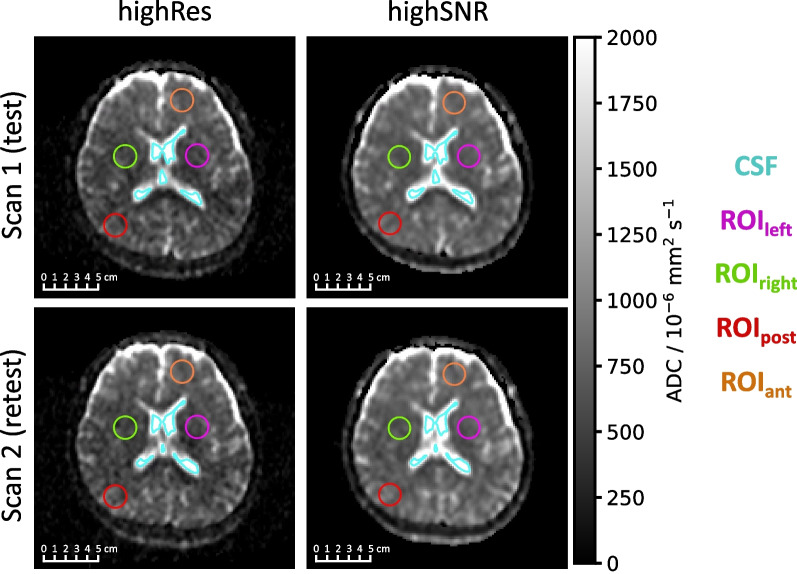
ADC maps with contours for evaluation. The same axial slice of the highRes (left column) and highSNR (right) variant are depicted for Scan 1 (top row) and Scan 2 (bottom) ADC maps for one volunteer. The contours considered for ADC assessment are overlaid. The depicted anatomy slightly differs between the four images due to the different slice thicknesses and positions between the two sequence variants and slightly different volunteer positioning before and after the break outside of the scanner

Figure 2 presents sample axial slices of the ADC maps for one volunteer for both Scan 1 and Scan 2 for both sequence variants. The CSF and four cylindrical ROIs within the brain tissue used for assessing ADCs are shown as contours. The median volume of the CSF contour among the ten volunteers was 4.8 cm^3^ (range 0.3–8.7 cm^3^). Median translations applied to the ADC maps of Scan 2 were below 1 mm for left–right (0.8 mm) and anterior–posterior (0.8 mm) directions, but larger in craniocaudal direction (2.7 mm). Median applied rotation angles were below 2° for pitch (1.4°), roll (1.5°), and yaw (1.6°).

The median duration of the break outside the scanner for the volunteers was 9.9 min (range 6.7–63.2 min). The measured ADCs (mean ± 1σ) for the five ROIs averaged over all volunteers, across the two scans and sequence variants are detailed in Table 3, alongside literature values. The mean ADCs were consistently larger for the highSNR compared to the highRes sequence variant for all investigated ROIs and both scans. Comparing to literature values, the mean ADCs, averaged over Scans 1 and 2, for the highSNR variant were within the given literature value ranges for all investigated ROIs. Conversely, for the highRes variant, the mean ADCs, averaged over Scans 1 and 2, were within literature value ranges for ROI_post_ and ROI_ant_, but smaller for the CSF, ROI_left_, and ROI_right_, with mean ADCs of (2261 ± 456) × 10^−6^ mm^2^/s (literature: 2730–3020 × 10^−6^ mm^2^/s) for the CSF, (530 ± 106) × 10^−6^ mm^2^/s (literature: 620–1090 × 10^−6^ mm^2^/s) for ROI_left_, and (517 ± 106) × 10^−6^ mm^2^/s (literature: 620–1090 × 10^−6^ mm^2^/s) for ROI_right_, respectively.

**Table 3:** Summary of volunteer results

ROI	highRes				highSNR				Literature
	Scan 1	Scan 2	RC	relRC	Scan 1	Scan 2	RC	relRC	
Unit	10^–6^ mm^2^/s	10^–6^ mm^2^/s	10^–6^ mm^2^/s	%	10^–6^ mm^2^/s	10^–6^ mm^2^/s	10^–6^ mm^2^/s	%	10^–6^ mm^2^/s
CSF	2264 ± 484	2258 ± 428	167 [117, 293]	7 [5, 12]	2868 ± 624	2878 ± 729	364 [254, 639]	12 [9, 22]	2730–3020
ROI_left_	528 ± 104	532 ± 107	62 [43, 108]	12 [8, 20]	680 ± 82	676 ± 82	41 [28, 71]	6 [4, 10]	620–1090
ROI_right_	518 ± 105	515 ± 106	66 [46, 116]	13 [9, 23]	673 ± 81	672 ± 80	46 [32, 80]	7 [5, 12]	620–1090
ROI_post_	668 ± 144	686 ± 139	70 [49, 123]	11 [8, 19]	816 ± 117	822 ± 122	64 [45, 112]	8 [5, 14]	620–1090
ROI_ant_	750 ± 123	748 ± 128	34 [24, 60]	5 [3, 8]	798 ± 92	804 ± 104	41 [29, 72]	5 [4, 9]	620–1090
All 4 cyl. ROIs			53 [44, 68]	9 [8, 12]			44 [36, 56]	6 [5, 7]	

The ADCs (mean ± 1σ) within each ROI, averaged over all ten volunteers are reported for both sequence variants and scans, alongside literature values [38]. Additionally, the RC and relRC values with their respective 95% confidence intervals are reported for each ROI separately, and for all four cylindrical ROIs combined

The RCs and relRCs for the volunteer cohort, calculated for all ROIs individually, and additionally for all four cylindrical ROIs combined, are listed in Table 3 for both sequence variants. For the CSF, a larger RC [95% CIs] was measured for the highSNR variant compared to the highRes variant, with values of 364 [254, 639] × 10^−6^ mm^2^/s and 167 [117, 293] × 10^−6^ mm^2^/s, respectively. This corresponds to relRCs [95% CIs] of 12% [9%, 22%] and 7% [5%, 12%], respectively. For the cylindrical ROIs individually, RCs within the range 34–70 × 10^−6^ mm^2^/s for highRes and 41–64 × 10^−6^ mm^2^/s for highSNR were measured, corresponding to relRCs ranges of 5–13% and 5–8%, respectively. For all four cylindrical ROIs combined, the RC and relRC were smaller for highSNR compared to highRes, with RCs [95% CIs] of 53 [44, 68] × 10^−6^ mm^2^/s for highRes and 44 [36, 56] × 10^−6^ mm^2^/s for highSNR, corresponding to relRCs [95% CIs] of 9% [8%, 12%] and 6% [5%, 7%], respectively.

**Fig. 3:**
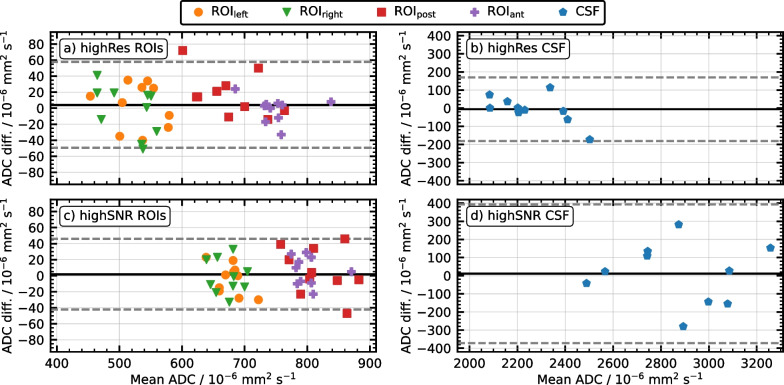
Bland–Altman plots for volunteers. The difference between the mean ADCs in Scan 1 and Scan 2 are plotted against their average for the highRes (**a**, **b**) and highSNR (**c**, **d**) variants, for the cylindrical ROIs within the brain tissue (**a**, **c**) and CSF (**b**, **d**), respectively. Note that subplots (**b**, **d**) have a different y-axis scale than subplots (**a**, **c**). The biases are shown as black solid lines and the LoAs at 95% confidence as dashed gray lines

Bland–Altman plots for the four cylindrical ROIs combined and the CSF are shown in Fig. 3. The observation based on the ADCs listed in Table 3, that generally smaller mean ADCs were measured for the highRes variant compared to the highSNR variant, is clearly reflected in the figure, where mean ADCs are consistently shifted towards higher values for the highSNR variant with respect to the highRes variant. While the LoAs bands were narrower for the highSNR compared to the highRes variant for the cylindrical ROIs, this was the other way around for the CSF. For the ROIs, the biases [LoAs at 95% confidence level] were + 4 [− 49, + 58] × 10^−6^ mm^2^/s for the highRes variant, and + 2 [− 42, + 46] × 10^−6^ mm^2^/s for the highSNR variant, respectively. For the CSF, they were − 6 [− 181, + 170] × 10^−6^ mm^2^/s (highRes) and + 11 [− 372, + 394] × 10^−6^ mm^2^/s (highSNR), respectively.

## Discussion

For both the diffusion phantom and the volunteers, both sequence variants under investigation exhibited very high repeatability after repositioning, assessed in a test–retest scheme. Absolute relative deviations between the scans before and after a break outside the scanner were less than 1% for water, DMSO, and PEG in the diffusion phantom, and relRCs [95% CIs] within four cylindrical ROIs within the brain were 9% [8%, 12%] for the highRes, and 6% [5%, 7%] for the highSNR sequence variant. The ADCs measured with the highSNR sequence variant were consistent with literature values for the volunteers, while for the diffusion phantom, smaller mean values were measured. In contrast, for the highRes sequence variant, for most ROIs, the ADCs were consistently smaller compared to literature values, indicating systematic underestimation of the true values. Overall, the highSNR sequence outperformed the highRes sequence in terms of ADC accuracy and repeatability, at the expense of an approximately doubled voxel volume, corresponding to an average voxel size increase by a factor of 1.25 in each spatial dimension. Hence, for accurate ADC measurements with high repeatability in a clinically reasonable scan time on a low-field MR-linac with the system’s head and neck coils, we recommend protocols with substantially increased voxel dimensions compared to high-field DWI protocols in order to compensate for the relatively low base SNR at 0.35 T.

This study primarily focused on assessing the repeatability, rather than the accuracy, of ADCs, with some literature values provided for reference. The ADCs measured for the diffusion phantom in this study were lower than reported literature values. The room temperature in this study (22.4 °C) was lower than in the cited study for comparison (24 °C) leading to different ADCs (e.g., for water, the expected theoretical value at 22.4 °C is 2150 × 10^−6^ mm^2^/s [43], which is closer to the measured value of about 2000 × 10^−6^ mm^2^/s of the highSNR sequence), thus, necessitating caution in interpreting the comparison with literature values. Despite this, the comparisons offered valuable insights.

The ADC for acetone in the diffusion phantom was lower than literature values by a factor of approximately 4–7. Acetone exhibited a signal intensity approximately 5 times weaker than water and 16 times weaker than PEG in the b0 image. Additionally, the high ADC posed challenges in accurate ADC reconstruction, since no acetone signal was detectable in the b500 or b800 images, as shown in Additional file 1: Fig. S1. Due to the monoexponential function used for fitting the noisy data, the derived ADCs for acetone were deemed unreliable [12, 44, 45]. However, this inaccuracy is less relevant for in vivo imaging since physiological ADCs are markedly lower than for acetone [38].

Comparisons with literature values for both the diffusion phantom and volunteers revealed underestimated ADCs for the highRes sequence variant. DWI at low-field MRI scanners is challenging due to inherently lower signal levels compared to higher field strength scanners. Our findings suggest that, for the given acquisition time, the spatial resolution of the highRes sequence variant (in-plane acquisition matrix resolution of 3.0 × 3.0 mm^2^ and a slice thickness of 5 mm) was too fine, resulting in underestimated ADCs. The SNR in the high b-value images for the highRes variant was low, as observed in the b800 image in Fig. 1, leading to underestimation of the true values [12, 29, 44, 45]. Potential solutions include excluding high b-value images, using an increasing number of averages for increasing b-values [12], extending the acquisition time, or incorporating a noise floor as an additional free parameter in ADC fitting, though the latter may increase the variance of derived ADCs. Nevertheless, the highSNR sequence demonstrated good ADC accuracy within a clinically acceptable acquisition time.

Repeatability is crucial for longitudinal studies in the context of MR-guided radiotherapy, where the goal is to monitor ADCs throughout the treatment for early response assessment [9, 33–35]. Both the diffusion phantom and volunteer scans exhibited high repeatability, despite some factors that introduced uncertainties that limited the achievable repeatability. This includes repositioning after the break outside the scanner, which relied solely on the laser positioning system. During registration of the pre- and post-break 3D-MRI datasets of the volunteers, median craniocaudal translations of 2.7 mm were applied to the ADC maps of Scan 2. Given the slice thicknesses of 5 mm or 7 mm of the two DWI sequence variants, this resulted in slice position offsets between the pre- and post-break ADC maps. Further uncertainties were introduced when interpolating the ROIs defined on the pre-break 3D-MRI dataset onto the image grids of the registered pre- and post-break ADC maps. These effects influenced the evaluation of ADCs within the ROIs, particularly for small CSF contours. Furthermore, no thermoplastic masks were employed, which would have reduced movements during DWI scanning. Therefore, repeatability assessments with patients in the clinical workflow, involving positioning based on image registration and couch shifts at the scanner and thermoplastic masks, may yield even higher repeatability of ADC maps [28, 37].

Weygand et al. reported on the long-term repeatability of ADCs derived from an EPI DWI pulse sequence measured within a NIST traceable diffusion phantom at a MRIdian MR-linac [31]. They achieved mean absolute deviations between the mean ADCs in different imaging session over 3 months of better than 2%, when averaged across all ROIs. In our study, absolute deviations of better than 1% were measured within the three vials within the range of physiological ADCs (water, DMSO, and PEG), albeit with measurements repeated after only a 7.5 min break. To the best of our knowledge, our study is the first to report on the in vivo repeatability of ADCs at the MRIdian MR-linac. However, systematic measurements of repeatability for phantoms and in vivo for different body sites have been conducted at the Elekta Unity MR-linac [9, 19, 39, 42, 46–48]. Lawrence et al. investigated in vivo repeatability in different ROIs within the brain [19]. They measured within-session (without repositioning) and between-session (on different days) repeatability coefficients [95% CIs] (converted from reported wSDs) of 18 [16, 22] × 10^−6^ mm^2^/s and 27 [25, 28] × 10^–6^ mm^2^/s for normal-appearing white matter, 18 [16, 22] × 10^–6^ mm^2^/s and 39 [36, 42] × 10^–6^ mm^2^/s for normal-appearing gray matter, and 125 [108, 150] × 10^–6^ mm^2^/s and 332 [305, 360] × 10^–6^ mm^2^/s for the CSF, respectively. Comparisons with the highSNR RCs of 44 [36, 56] × 10^–6^ mm^2^/s for cylindrical ROIs within the brain and 364 [254, 639] × 10^–6^ mm^2^/s for the CSF measured in our study, reveal lower RCs and narrower CIs in their study, potentially also attributable to a larger patient cohort, the use of a thermoplastic mask, and a more accurate repositioning system. However, caution is warranted when comparing repeatability values among different studies due to differences in scanners, employed DWI pulse sequence parameters, diffusion phantoms or body sites, and break durations between test and retest scans. Furthermore, the RC calculations are not always appropriately adjusted for small sample sizes, particularly for phantom studies.

If changes between measurements exceed the RC, these changes are, with 95% confidence, caused by a true biomarker change rather than mere measurement uncertainties [40]. Changes in ADCs within the GTV in high-grade gliomas throughout radiotherapy have been reported to range up to 20%, with interquartile ranges of [− 7.5%, 7.8%] within 13 patients at the end of radiotherapy (after 6 weeks) [19]. We measured a relRC of 6% [5%, 7%] when considering all cylindrical ROIs within the brain tissue. Despite the limited number of patients in the study by Lawrence et al., the comparison with our relRCs underscores the potential of DWI at low-field MR-linacs in detecting true tumor ADC changes throughout radiotherapy for early treatment response assessment.

Some other study limitations must be kept in mind. First, the spatial resolutions of the investigated DWI sequence variants were constrained compared to scans at higher field strengths, owing to inherent signal limitations stemming from the low magnetic field strength of 0.35 T. Additionally, an evaluation of the geometric accuracy of the ADC maps, which would require a dedicated distortion phantom, was beyond the scope of this study. However, previous research by Weygand et al. demonstrated submillimeter geometric accuracy of ADC maps reconstructed from the same sequence, although with different sequence parameters and only system-dependent geometric distortions considered [31]. Lastly, intravoxel incoherent motion (IVIM) effects at low b-values were neglected in our study [12].

Looking ahead, our study has provided valuable insights into the repeatability of the investigated ADC measurements with two different DWI pulse sequence variants. Further steps towards clinical implementation would involve imaging studies for brain cancer patients. With the single-shot EPI-based DWI pulse sequence having been assessed for the brain in this study, and sarcomas in the study by Weygand et al. [31], the sequence could be adapted and evaluated for use in other body sites, such as prostate, rectum, and head and neck cancer [2].

## Conclusions

In conclusion, our study evaluated for the first time the in vivo repeatability of ADC measurements with a single-shot EPI DWI pulse sequence on a low-field MR-linac. The investigation focused on two sequence variants, highRes and highSNR, emphasizing spatial resolution and signal-to-noise ratio, respectively. Both variants demonstrated high repeatability for a diffusion phantom and brains of ten volunteers, with the highSNR sequence outperforming the highRes in terms of both accuracy and repeatability. The high in vivo repeatability observed in this study confirms the potential utility of DWI at low-field MR-linacs for early treatment assessment and biologically-guided radiotherapy.

### Supplementary Information

**Additional file 1: Fig. S1.** Exemplary diffusion-weighted images and ADC maps for the diffusion phantom to support the observations regarding the diffusion phantom results, discussed in the Discussion section.

## Data Availability

The datasets used and/or analyzed during the current study are available from the corresponding author on reasonable request.

## Abbreviations

ADC: Apparent diffusion coefficient
AP: Anterior–posterior
bSSFP: Balanced steady-state free precession
CI: Confidence interval
CSF: Cerebrospinal fluid
df: Degrees of freedom
DMSO: Dimethyl sulfoxide
DWI: Diffusion-weighted imaging
EPI: Echo-planar imaging
highRes: High resolution
highSNR: High SNR
IVIM: Intravoxel incoherent motion
LoAs: Limits of agreement
LR: Left–right
MRI: Magnetic resonance imaging
PEG: Polyethylene glycol
QIBA: Quantitative Imaging Biomarkers Alliance
RC: Repeatability coefficient
relRC: Relative repeatability coefficient
ROI: Region-of-interest
SI: Superior-inferior
SNR: Signal-to-noise ratio
TE: Echo time
TR: Repetition time
TSE: Turbo spin echo
