# Supplementary material for: Repeatability quantification of brain diffusion-weighted imaging for future clinical implementation at a low-field MR-linac
Source: Radiat Oncol. 2024 Mar 6;19:31. doi: 10.1186/s13014-024-02424-7 (PMC10916154; doi:10.1186/s13014-024-02424-7)
Supplement: Supplementary file 1 — Additional file 1: Fig. S1. Exemplary diffusion-weighted images and ADC maps for the diffusion phantom to support the observations regarding the diffusion phantom results, discussed in the Discussion section. [file 13014_2024_2424_MOESM1_ESM.pdf]

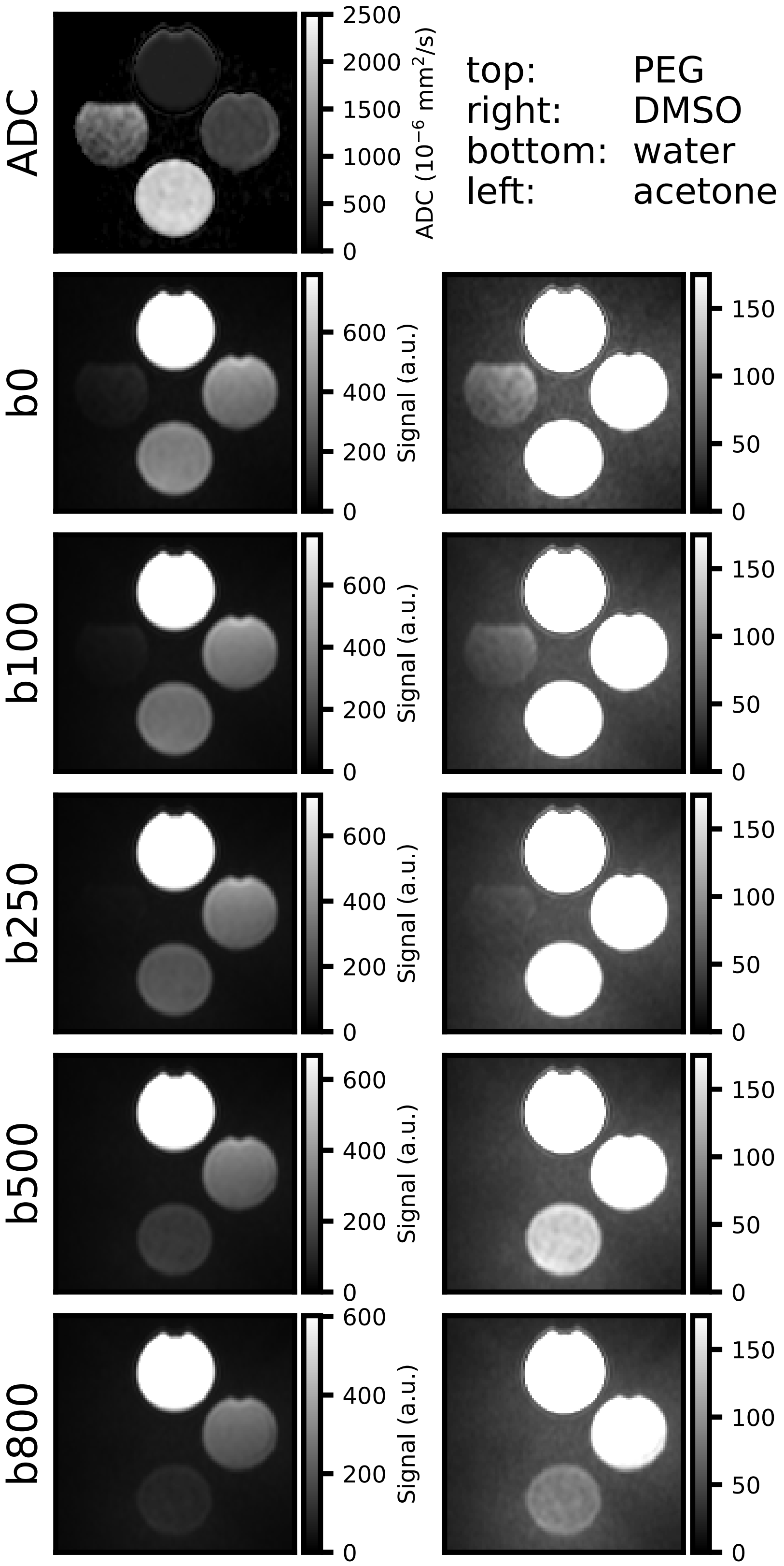

Supplementary Figure 1: ADC and diffusion-weighted images for diffusion phantom. The ADC map (top left) and diffusion-weighted images at different b-values (rows 2-5) are shown for an axial slice through the four vials of the diffusion phantom (top: PEG, right: DMSO, bottom: water, left: acetone) for the highSNR sequence variant (Scan 1; before break). The left column shows the diffusion-weighted images at different b-values with variable window/level settings, while the window/level settings for the images in the right column were fixed to maximize contrast for very low signal levels. The images were cropped for better visibility. The signal for acetone is not distinguishable from background noise in the b500 and b800 images, due to its low signal and high diffusion coefficient, resulting in the underestimated ADCs reported in the study.
